# Mortality Trends in Women and Men Presenting with Acute Coronary Syndrome: Insights from a 20-Year Registry

**DOI:** 10.1371/journal.pone.0070066

**Published:** 2013-07-31

**Authors:** Ayman El-Menyar, Emad Ahmed, Hajar Albinali, Hassan Al-Thani, Abdurrazak Gehani, Rajvir Singh, Jassim Al Suwaidi

**Affiliations:** 1 Department of Clinical Medicine, Weill Cornell Medical School, Doha, Qatar; 2 Department of Trauma Surgery, Clinical Research, Hamad Medical Corporation, Doha, Qatar; 3 Cardiology Unit, Department of Medicine, Ahmed Maher Teaching Hospital, Cairo, Egypt; 4 Department of Cardiology, Heart Hospital, Hamad Medical Corporation, Doha, Qatar; 5 Department of Vascular Surgery, Hamad Medical Corporation, Doha, Qatar; Virginia Commonwealth University, United States of America

## Abstract

**Background:**

Coronary artery disease (CAD) is the leading cause of mortality worldwide. The present study evaluated the impact of gender in patients hospitalized with acute coronary syndromes (ACS) over a 20-year period in Qatar.

**Methods:**

Data were collected retrospectively from the registry of the department of cardiology for all patients admitted with ACS during the study period (1991–2010) and were analyzed according to gender.

**Results:**

Among 16,736 patients who were admitted with ACS, 14262 (85%) were men and 2474 (15%) were women. Cardiovascular risk factors were more prevalent among women in comparison to men. On admission, women presented mainly with non-ST-elevation ACS and were more likely to be undertreated with β-blockers (BB), antiplatelet agents and reperfusion therapy in comparison to men. However, from 1999 through 2010, the use of aspirin, angiotensin-converting enzyme inhibitors and BB increased from 66% to 79%, 27% to 41% and 17% to 49%, respectively in women. In the same period, relative risk reduction for mortality was 64% in women and 51% in men. Across the 20-year period, the mortality rate decreased from 27% to 7% among the Middle Eastern Arab women. Multivariate logistic regression analysis showed that female gender was independent predictor of in-hospital mortality (odd ratio 1.51, 95% CI 1.27–1.79).

**Conclusions:**

Women presenting with ACS are high-risk population and their in-hospital mortality remains higher for all age groups in comparison to men. Although, substantial improvement in the hospital outcome has been observed, guidelines adherence and improvement in the hospital care have not yet been optimized.

## Introduction

Traditional cardiovascular risk factors are overall similar for women and men across various regions of the world [1]. With advances in diagnosis and management of acute coronary syndrome (ACS), the cardiovascular mortality in men has been reduced over the past decade. However, the mortality rate among women has continued to increase every year since 1984 [2]. Previous data showed that women with ACS were less likely to undergo diagnostic and therapeutic procedures compared to men [3], [4]. Poon et al, reported an overall temporal increase in the use of invasive cardiac procedures, however, women with ACS were more likely to be treated conservatively when compared with men [5]. Underestimation of risk among women presenting with ACS by the treating physician may be an important reason. Indeed, gender-related discrepancy in treatment strategies has been observed in several trials and registries [6]–[8].

Several hypotheses have been postulated regarding the gender-related disparities for ACS treatment. These include higher prevalence of atypical presentations and vasospastic disease in women, which could make their diagnosis and subsequent management less feasible [6], [9]. Overall, women have less obstructive coronary artery disease (CAD) than men regardless of the ACS type and age [10]–[12]. Furthermore, physicians may not recommend coronary interventions in women because of the presumed increased risks when compared to men and the doubtful potential benefits if coronary obstructive lesions are not confirmed [13]–[15]. Recently, the European Society of Cardiology guidelines recommended early coronary intervention within the first 24 hrs for high-risk patients presented with non-ST-segment elevation ACS regardless of gender [16].

Generally, data from different countries have reported worse outcomes in women presenting with ACS compared to men [8], [17]–[18]. In the current study, we evaluate the impact of gender on the trends, clinical presentation, management and in-hospital mortality in a large sample of patients hospitalized with ACS across a 20-year period.

## Methods

The Cardiology and Cardiovascular Surgery Database at Hamad Medical Corporation (HMC) in Qatar was used for this study. This hospital provides in-patient and out-patient tertiary care for the residents of Qatar regardless of ethnicity. More than 95% of cardiac patients in Qatar are treated at HMC which makes it an ideal center for population-based studies. A case report form with a specific registration number for each cardiac patient admitted to the cardiology department was filled by the assigned physician. Case reports were filled using standard definitions and completed before the patient’s hospital discharge. Data were collected according to predefined criteria for each variable. These records have been coded and registered electronically. Ten percent randomly selected records from the data were checked by an independent physician for its accuracy before feeding and data analysis. With the described database, all patients admitted with ACS in the 20-year period between January 1991 and end of 2010 were retrospectively reviewed. Data registered into a computer by a data entry operator were randomly cross checked by the physician at the cardiology department. Transferred patients or patients with missing data were excluded from the study. Ethical approval was obtained from the research committee at HMC before data collection and analysis. The ethics committee waived the need of informed consent for the study because of its retrospective nature and data were analyzed anonymously.

### Definition

Diagnosis of different types of ACS and definitions of data variables were based on the American College of Cardiology (ACC) clinical data standards [19]. Use of adjunct therapy during hospitalization was recorded for every patient. Cardiovascular risk factors were defined previously [20]–[21]. The presence of diabetes mellitus (DM) was determined by the previous or current medical record of the patient reporting the diagnosis of DM that had been treated with oral medications or insulin. The presence of dyslipidemia was determined by the demonstration of a fasting cholesterol >5.2 mmol/L in the patient’s medical record, or any history of treatment for dyslipidemia. The presence of hypertension (HTN) was determined by documentation of HTN in the medical record or if the patient was on regular antihypertensive medications. Smoking history: patients were divided into current cigarette smokers, past smokers (defined as ≥6 months abstinence from smoking) and those who never smoked.

### Statistical Analysis

Data were presented as proportions, mean ± standard deviation (SD) or median (range) as appropriate. Baseline demographics, past medical history, clinical presentation, medical therapy, cardiac procedures and clinical outcomes were compared between men and women. Data were also analyzed according to gender in different age groups (<50, 51–70 and >70 years) and across every 4-year period from (1991–1994) to (2007–2010). Statistical analyses were conducted using Student-t test and One Way ANOVA, wherever applicable, for continuous variables and Pearson chi-square (χ^2^) test for categorical variables. Variables influencing in-hospital mortality were also analyzed using multiple logistic regressions analysis. Adjusted Odds Ratios (OR), 95% confidence interval (CI), and p values were reported for significant predictors. All p values were the results of 2-tailed tests and values <0.05 were considered significant. Statistical Package for Social Sciences (SPSS) version 19.0 has been used for the analysis.

## Results

**Figure 1:**
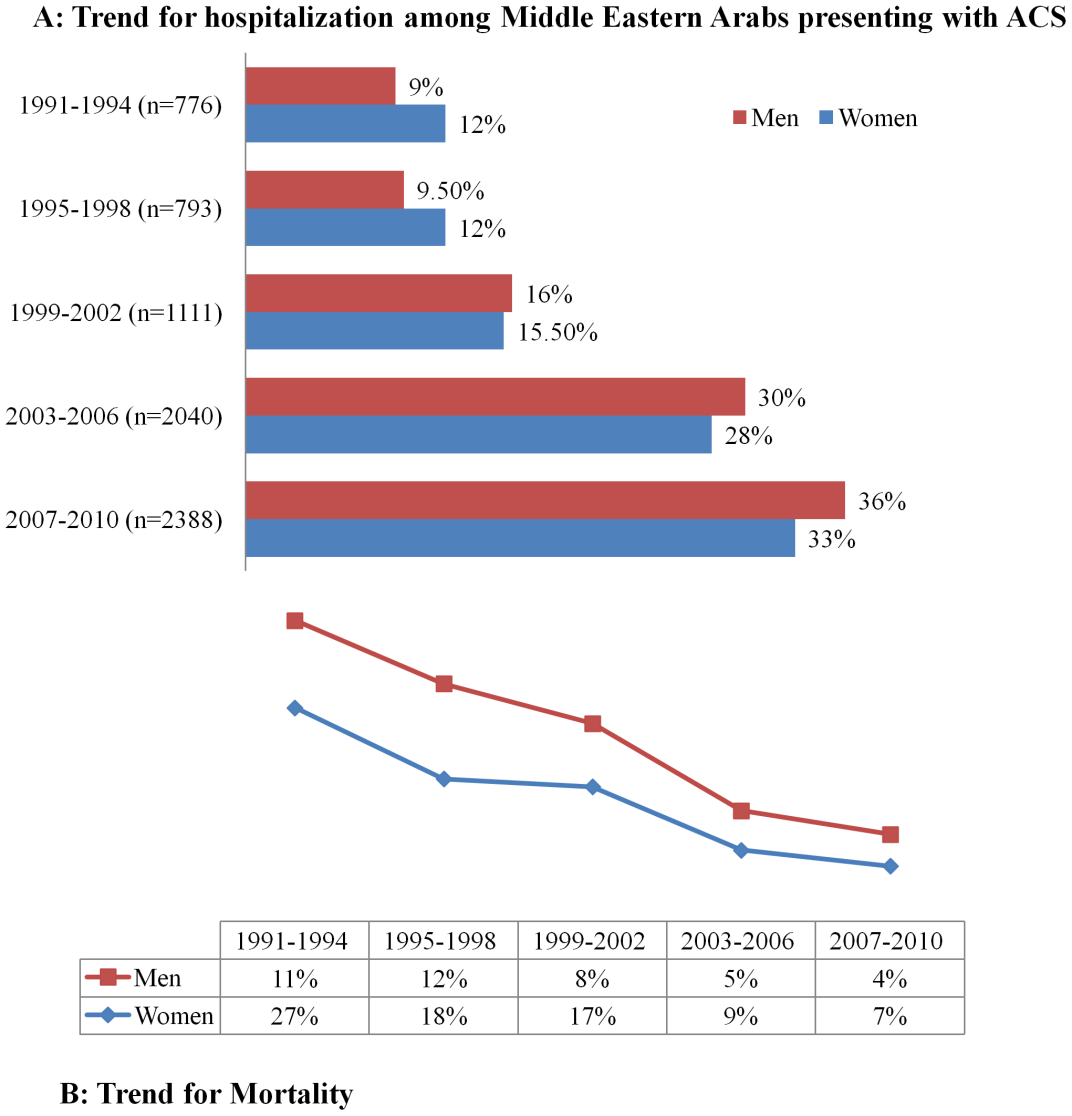
Acute coronary syndrome among Middle Eastern Arab (Men and Women): A: Trend for hospitalization. B: Trend for mortality.

Between 1991 to the end of 2010, a total of 16,736 patients were admitted with ACS; of them 14,262 (85%) were men and 2,474 (15%) were women. Overall, 7,200 (43%) patients presented with ST-elevation MI (STEMI), 5572 (33%) patients with Non-ST elevation MI (NSTEMI) and 3964 (24%) patients with unstable angina (UA). Over the 20-year period, there was an increase in the total number of Arab patients hospitalized with ACS regardless of gender (Figure 1A).

Table 1 shows the clinical presentation and baseline characteristics of patients stratified by gender. Women were more likely to have HTN, DM, renal failure, dyslipidemia, prior MI and CABG. Whereas, the incidence of current smoking and prior PCI were higher in men (P = 0.001 for each). Women were more likely to present with atypical symptoms including higher frequency of dyspnea compared to men (33% vs. 19%, P = 0.001). Men had significantly higher level of mean serum triglyceride and low-density lipoprotein (P = 0.001 for each), and lower levels of high-density lipoprotein (P = 0.001) in comparison to women.

**Table 1:** Patients’ characteristics, co-morbidities and outcomes according to gender.

	Women	Men	P-value
**Number of patients**	2474 (14.8)	14262 (85.2)	
**Age** (mean±SD)	62±12	53±11	0.001
**Race**			
Middle-Eastern Arabs	1892 (76.5)	5216 (36.6)	0.001
South Asians	321 (13)	7164 (50.2)	
Others	261 (10.5)	1882 (13.2)	
**Cardiovascular risk factors**			
Hypertension	1684 (68.1)	5210 (36.5)	0.001
Diabetes mellitus	1626 (65.7)	5333 (37.4)	0.001
Current smoker	89 (3.6)	5497 (38.5)	0.001
Chronic renal impairment	164 (6.6)	397 (2.8)	0.001
Dyslipidemia	672 (27.2)	2882 (20.2)	0.001
Family history of CAD	146 (1.5)	645 (2.0)	0.001
Prior myocardial infarction	484 (19.6)	2306 (16.2)	0.001
Prior coronary artery bypass grafting	123 (5)	570 (4)	0.03
Prior PCI	190 (7.7)	1546 (10.8)	0.001
**Total cholesterol** *(mmol/L)* (mean±SD)	4.9±1.4	4.9±1.3	0.23
**Serum triglyceride** *(mmol/L)* (mean±SD)	1.8±1.14	1.9±1.18	0.001
**Low density lipoprotein** *(mmol/L)* (mean±SD)	2.6±1.04	2.8±1.09	0.001
**High density lipoprotein** *(mmol/L)* (mean±SD)	1.17±0.38	0.98±0.31	0.001
**Troponin T** *(ng/ml)*	1336 (13.7)	7414 (23.4)	0.001
**CK-MB** *(u/L)* (mean±SD)	75±430	148±623	0.001
**Atypical chest pain**	542 (5.6)	1760 (5.6)	1.00
**Ischemic chest pain**	1248 (12.8)	4950 (15.6)	0.001
**Shortness of breath**	3210 (32.9)	6149 (19.4)	0.001
**Left ventricular ejection fraction**	45.8	42.9	0.001
**Type of ACS**			
ST-elevation MI	604 (24.4)	6596 (46.2)	0.001
No-ST-elevation MI	993 (40)	4579 (32)	
Unstable angina	877 (35)	3087 (22)	
**Stroke**	20 (0.8)	37 (0.3)	0.001
**Death**	262 (10.6)	701(4.9)	0.001
**Year of Admission**			
1991–94	202 (8.2)	1573 (11)	0.001
1995–98	211 (8.5)	1625 (11.4)	
1999–02	384 (15.5)	2028 (14.2)	
2003–06	753 (30.4)	3899 (27.3)	
2007–10	924 (37.3)	5137 (36)	

CAD: coronary artery disease, PCI: percutaneous coronary intervention, ACS: acute coronary syndrome; All values in parenthesis are percentages.

### Gender and Trends of the Cardiovascular Risk Factors

**Figure 2:**
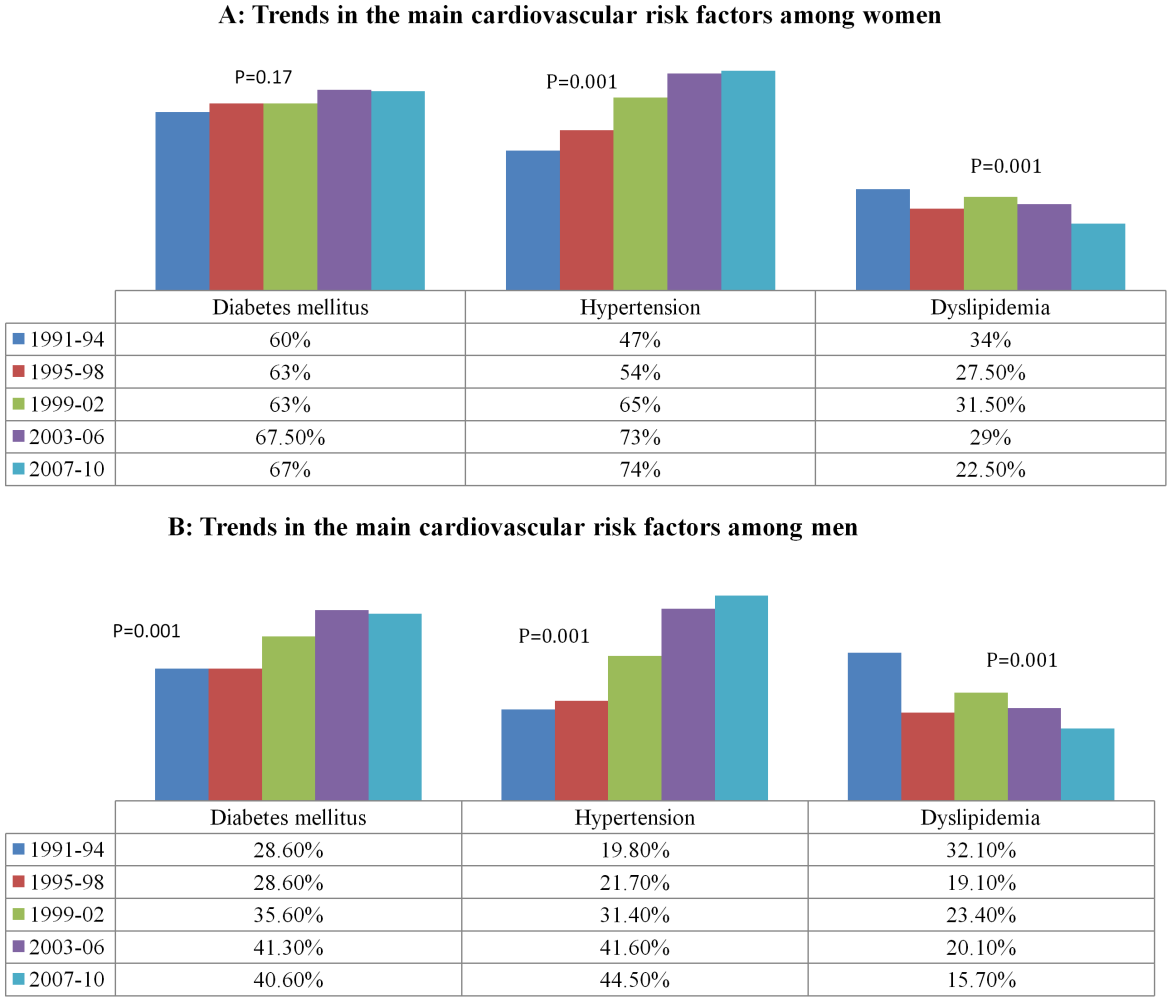
Trend of cardiovascular risk factors in women (A) and men (B) across 20 years.

Figure 2A shows an increased rate of hypertension while the rate of dyslipidemia decreased among women over the 20-year study period (p = 0.001). Also, an increasing trend in the rate of DM has been observed but was not statistically significant (p = 0.17). Among men, there was an increase in the frequency of DM and hypertension and a decrease in the rate of dyslipidemia (p = 0.001 for all) (Figure 2B).

### Age and Gender

The mean age of patients was 54±11.9 years; 41% of patients were ≤50 years old, 49% were between 51 and 70 years and 10% were above 70 years of age. Seventeen percent of women presenting with ACS were ≤50 years old, 61% were between 51–70 years and 22% were above age of 70. Eight percent of men were older than 70 years, whereas 92% were almost equally distributed in age groups ≤50 and 51–70 years.

### Age, Gender and Type of ACS

Overall, more men presented with STEMI when compared to women **(**46% vs. 24%). In comparison to men, women were frequently presented with NSTEMI (40% vs. 32%; P = 0.001) and UA (35% vs. 22%; P = 0.001) (Table 2). The majority of women under age of 50 presented mainly with unstable angina, while the majority of women older than 70 years of age presented with STEMI. Between the age of 51 and 70, both unstable angina and NSTEMI equally dominated.

**Table 2:** Type of coronary syndrome and mortality in women and men at different age group.

Variables	Overall		Age ≤50 yrs			Age 51–70 yrs			Age >70 yrs		
	Women	Men	Women	Men	P value	Women	Men	P value	Women	Men	P value
**N (%)**	2474(15)	14262(85)	410 (5.9)	6528 (94.1)		1510 (18.5)	6642 (81.5)		553 (33.7)	1087 (66.3)	
**STMI**	604 (24)	6596 (46)	117 (28.5)	3576 (34.8)	0.001	368 (24.4)	2729 (41.1)	0.001	119 (21.5)	288 (26.5)	0.001
**NSTMI**	993 (41)	4579 (32)	121 (29.5)	1747 (26.8)		593 (39.3)	2322 (35)		278 (50.3)	509 (46.8)	
**UA**	877 (35)	3087 (22)	172 (42)	1205 (18.5)		549 (36.4)	1591 (24)		156 (28.2)	290 (26.7)	
**Death**	11%	5%	30 (7.3)	201 (3.1)	0.001	136 (9.0)	358 (5.4)	0.001	95 (17.2)	142 (13.1)	0.001

STEMI: ST-Elevation Myocardial Infarction; NSTMI: Non ST-Elevation Myocardial Infarction; UA: Unstable Angina, All values in parenthesis are percentages.

Men younger than 50 years of age presented mainly with STEMI, whereas the majority of men older than 70 years of age presented with NSTEMI. Between the age of 51 and 70, unstable angina was the predominant presentation among men admitted with ACS.

### Management

**Figure 3:**
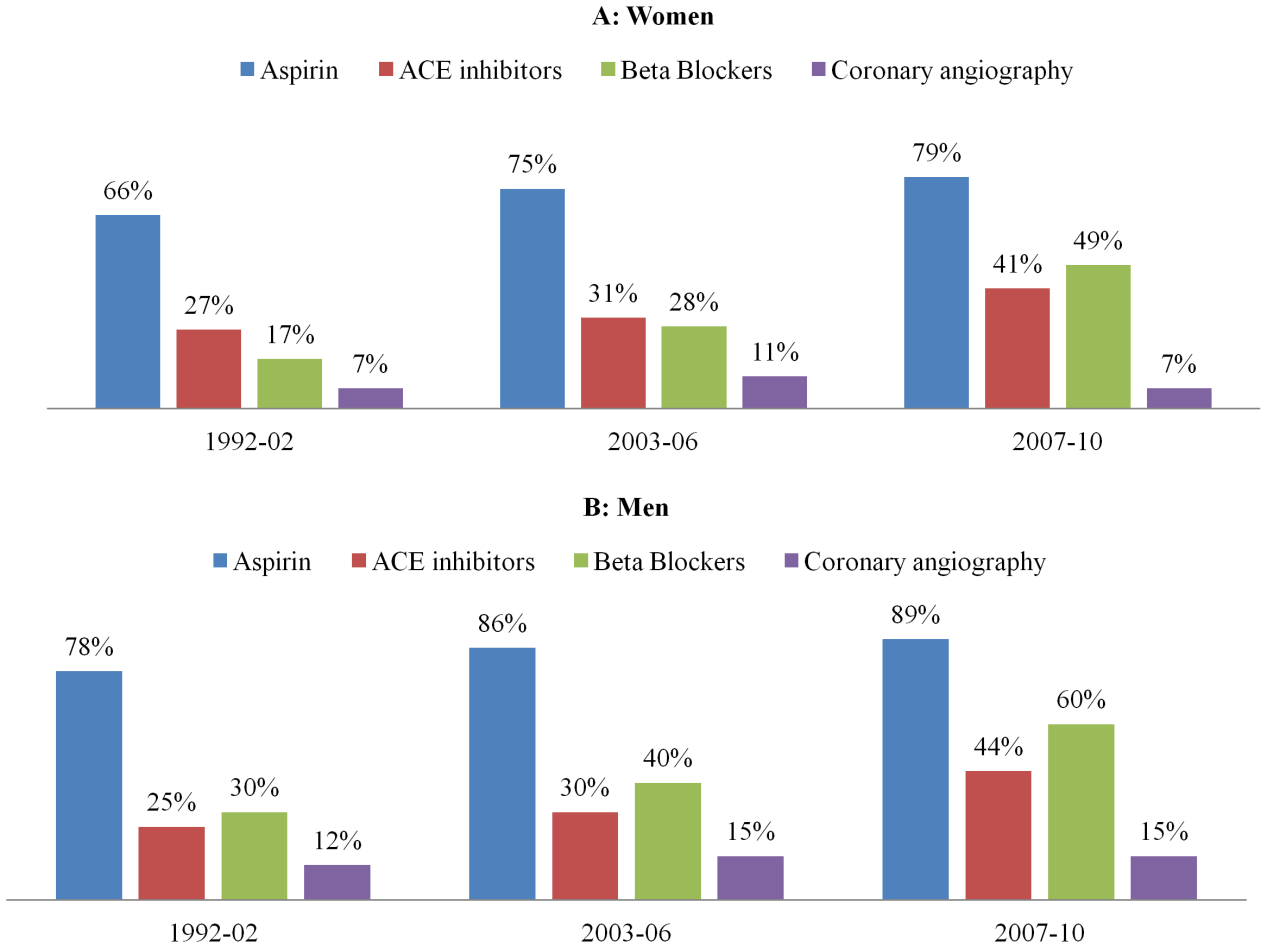
Trend of treatment in Women (A) and Men (B).

- ***Pre-admission therapy*** **:** In comparison to men, prior to the index admission, women were more likely to be on β-blockers (BB), calcium channel blockers (CCB), angiotensin-converting enzyme (ACE) inhibitors/angiotensinogen receptor blockers and antiplatelet therapy (Table 3).
- ***On admission*** **:** Women were less likely to receive BB, antiplatelet therapies, unfractionated heparin, and thrombolytic therapy. The uses of CCB, ACE inhibitors and low molecular weight heparin were more common in women. Women were less likely to undergo coronary angiography (8% vs. 13%, P = 0.001) and PCI (5% vs. 7%, P = 0.002).
- ***Admission medication based on patients’ age:*** In age group≤50 years, women were less likely to receive antiplatelet (89% vs. 93%, P = 0.001) and BB therapies (48.5% vs. 55%, P = 0.01) in comparison to men, while ACE inhibitors were more frequently prescribed to women (33.4% vs. 25.5%, P = 0.001). In age group between 51–70 years, women were less likely to receive antiplatelet agents (89% vs. 92%, p = 0.001), and BB (41.5% vs. 49%, p = 0.001). While in age group>70 years, there was no significant difference between women and men regarding admission medications except for higher percent of BB in men (P = 0.04).
- ***At discharge*** **:** Women were less likely to be discharged on BB, and antiplatelet agents (P = 0.001). On the other hand, women were more likely to be treated with CCB (P = 0.001). There was no difference in the treatment with statin in both sexes (P = 0.90).
- ***In-hospital management Trend:*** Figure 3 (A and B) demonstrates the treatment trend for men and women across the period from 1999 to 2010. Prescription of antiplatelet therapies increased from 66% to 79% in women and from 78% to 89% in men. The use of BB and ACE inhibitors increased from 17% to 49% and 27% to 41%, respectively in women and from 30% to 60% and 25 to 44%, respectively in men. However, coronary interventions were lower in women compared to men.

**Table 3:** Treatment according to gender.

	Women	Men	P-value
**Pre-admission medications**			
β-blockers	454 (18.4)	1835 (12.9)	0.001
Calcium channel blockers	239 (9.7)	515 (3.6)	0.001
ACE/ARB	447 (18.1)	1413 (9.9)	0.001
Anti-platelet agents	1239 (50.1)	4299 (30.1)	0.001
**On Admission**			
β-blockers	1006 (40.7)	7253 (50.9)	0.001
Calcium channel blockers	404 (16.3)	1007 (7.1)	0.001
ACE/ARB	840 (34)	4326 (30.3)	0.001
Anti-platelet agents	2264 (91.5)	13483 (94.5)	0.001
Heparin	723 (29)	5687 (40)	0.001
LMW Heparin	535 (22)	2630 (18)	0.001
Glycoprotein blocker	77 (3)	668 (5)	0.001
**Thrombolysis**	285 (11.5)	4469 (31)	0.001
**Coronary angiography**	192 (8)	1837 (13)	0.001
**PTCA**	130 (5)	999 (7)	0.002
**Stent**	96 (4)	655 (5)	0.11
**At Discharge**			
β-blockers	577 (23.3)	4547 (31.9)	0.001
Calcium channel blockers	539 (21.8)	1456 (10.2)	0.001
Diuretics	756 (30.6)	2232 (15.6)	0.001
ACE/ARB	993 (40.1)	5722 (40.1)	0.98
Anti-platelet agents	2046 (82.7)	13019 (91.3)	0.001
Clopidogerl	769 (31.1)	4981 (34.9)	0.001
Aspirin	2007 (81.1)	12899 (90.4)	0.001
Statin	1284 (52)	7381 (52)	0.90

ACE/ARB: Angiotensin converting enzyme inhibitor; PTCA: Percutaneous Coronary Angioplasty; All values in parenthesis are percentages.

### In-hospital Mortality Rate and Trend

**Figure 4:**
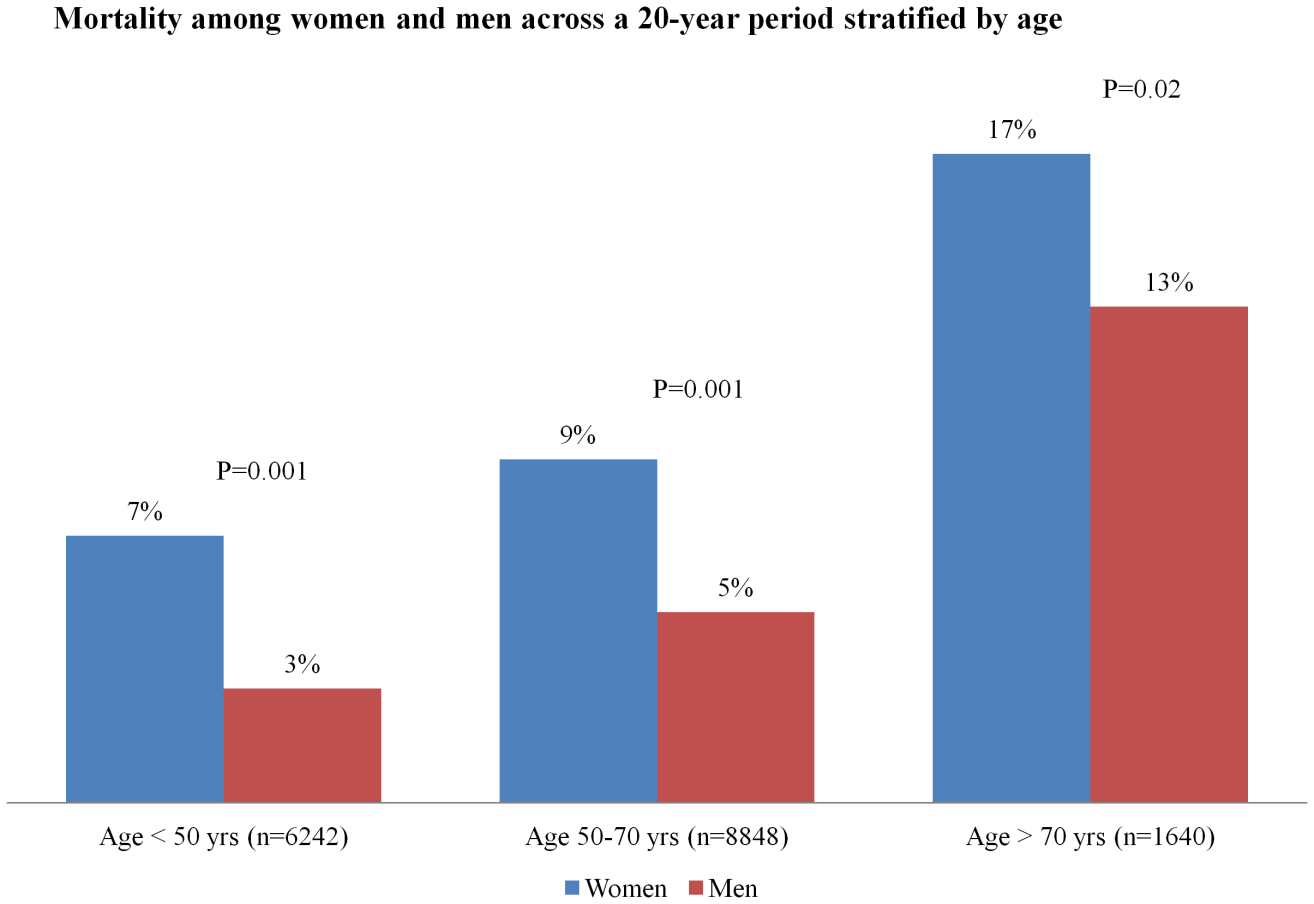
Mortality rate in men and women stratified by age.

Overall in-hospital mortality rate across the 20-year period was higher in women when compared to men (10.6% vs. 4.9%, P = 0.001). Among younger patients (<50 years), the rate of death in women was higher (7.3% vs. 3.1%, P = 0.001) and this trend for high mortality was also observed among women of age between 51–70 years (9% vs. 5.4%, p = 0.001) and above 70 years of age (17.2% vs. 13.1%, P = 0.02) in comparison to men (Figure 4).

**Figure 5:**
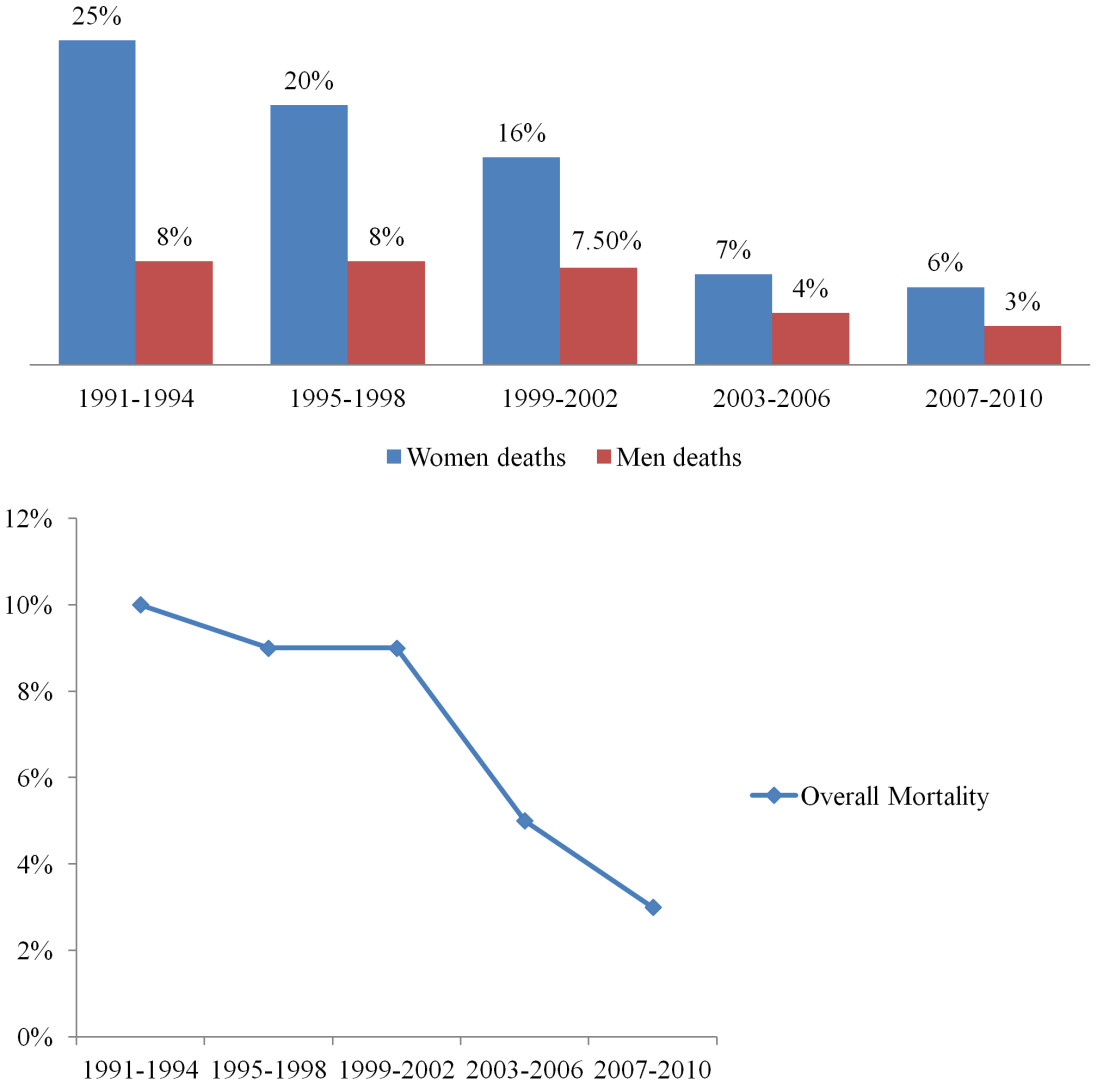
Overall and trend of mortality in men and women.

Figure 1B shows the trend of mortality in the Middle Eastern Arab women and men hospitalized with ACS**.** The overall in-hospital mortality rate was significantly decreased among Arab women from 27% to 6.6% between the periods (1991 to 1994) and (2007 to 2010), respectively. Relative risk reduction (RRR) in mortality between 1991–2002 and 2003–2010 was 64% and 51% in women and men, respectively. Figure 5 demonstrates the overall and trend for in-hospital mortality across 20 years in men and women.

### Multiple logistic regression analysis

Female gender was found to be an independent predictor (OR 1.51; 95% CI 1.27–1.79, P = 0.001) for in-hospital mortality after adjusting other variables such as age, DM, hypertension, dyslipidemia, current smoking, chronic renal impairment, prior MI, type of ACS and year of admission (Table 4).

**Table 4:** Multivariate regression analysis for predictors of in-hospital mortality.

Variable	Adjusted OR	95% CI	P-value
**Age**	1.04	1.03–1.05	0.001
**Female**	1.80	1.48–2.12	0.001
**Diabetes Mellitus**	1.60	1.38–1.86	0.001
**Hypertension**	0.91	0.78–1.07	0.25
**Dyslipidemia**	0.66	0.55–0.80	0.001
**Chronic renal impairment**	2.40	0.78–3.23	0.001
**Current smoking**	0.75	0.62–0.90	0.002
**Prior myocardial infarction**	1.61	1.35–1.91	0.001
**Diagnosis**			
Unstable angina	1	–	–
Non-ST-elevation MI	19.38	13.2–28.5	0.001
ST-elevation MI	6.53	4.44–9.60	0.001
**Year of Admission**			
1991–94	1	–	–
1995–98	1.17	0.91–1.50	0.23
1999–02	1.64	1.29–2.09	0.001
2003–06	0.90	0.70–1.14	0.37
2007–10	0.72	0.56–0.93	0.01

Adjusted OR: Adjusted Odd Ratio; 95% CI: 95% Confidence Interval.

## Discussion

The present study reports a large data of patients hospitalized with ACS over 20 years in Qatar. According to 2010 census, Qatar has a population of about 1.7 million with female to male ratio of 1∶3. Our study demonstrates important differences between women and men presenting with ACS. There are several key findings in this study. ***First***, female gender was independent predictor of in-hospital mortality in patients presenting with ACS. The current analysis shows that women had higher mortality rates compared to men in all age groups. However, data from US National Registry of Myocardial Infarction-2 found an increased mortality risk for young women compared with young men, with no mortality difference in the older population [22].

***Second***, gender differences exist in many aspects of ACS; including presentation, diagnosis, treatment and outcomes. ***Third***, over the study period there was a substantial increase in the number of patients admitted with ACS regardless of gender. Furthermore, the rate of ACS admissions from (1991–1994) to (2007–2010) increased four times among Arab women patients and seven times among South Asian patients. ***Fourth***, although women were less likely to be prescribed evidence based medications, there were upward trends in the use of aspirin, BB, ACE inhibitors across the study period regardless of gender. The period between 1999 through 2010 showed improvement in the utilization of evidence-based therapies in our institution, although the use of early invasive therapy remained low. ***Fifth***, the current study reports a significant reduction of the in-hospital mortality rate among ACS women and men, and this improvement in outcome most likely attributes to the increased use of evidence-based therapies. Similarly, Lundblad et al [23] analyzed MI patients in Northern Sweden across a 20-year period (1985–2004) and reported a mortality reduction of 69% and 45% in men and women, respectively. This improvement in outcome was relatively better for women between 1991 and 2010 in our study. ***Sixth***, the present report demonstrates differential trends in the cardiovascular risk factors across 20 years in men and women. Generally, women were more likely older and had greater cardiovascular risk factors compared to men. The mean age of patients in the current registry is comparable to the CREATE registry from India and almost a decade younger than those reported from developed countries [24]–[28].

The diagnosis of ACS in women is more challenging especially when it is based on history alone [29]–[31]. Women are more likely to present with atypical symptoms including higher frequency of dyspnea when compared to men; a factor that increases the likelihood of misdiagnosis among women. These atypical presentations could be attributed to difference in anatomy and pathophysiology of CAD in women compared to men which needs high index of suspicion for the prompt diagnosis of ACS in women [28].

Serum troponin is the biochemical marker of choice for evaluating the acute risk of patients with ACS without persistent ST elevation and to guide further management [32]. In our study, the prevalence of positive troponin T and mean CK-MB level were significantly lower in women compared to men. Complete plaque rupture is the frequent pathology in ACS men that leads to more severe stimulus for repetitive thrombus embolization with consecutive troponin release [33]. On the other hand, plaque erosion is more common in women. According to the Universal Classification of MI, spontaneous MI (MI type 1) that occurs due to atherosclerotic plaque rupture, ulceration, fissuring, erosion, or dissection were found particularly in women without angiographic evidence of severe CAD [34].

Moreover, along with previous registries, our findings show that females with ACS without ST elevation MI are more likely to have higher Killip class than their male counterparts [5]. In terms of ACS types, the present study demonstrates that the proportion of women with STEMI is significantly lower than that of men, whereas the proportion of women with unstable angina and NSTEMI is significantly greater when compared to men. However, the influence of age at the time of presentation should be taken into consideration i.e., women younger than 50 years of age are more likely to have unstable angina whereas; women older than 70 years of age are mainly presented with STEMI. This finding could be related to the pathophysiology of the vulnerable plaques (i.e., erosion vs. rupture) in each gender among young versus older age. Also, discrepancy between both sexes in the ACS may attribute to the differences in thrombotic and fibrinolytic activity, variability in the extent and severity of coronary lesions and presence of collateral blood flow [35]–[39].

Despite the fact that women with ACS represent higher risk group compared to men, they are undertreated with evidence-based therapies. Previous studies showed that both acute and discharge therapies according to guidelines were applied less frequently to women who were treated mainly with conservative management [3], [6], [7], [40]–[43]. Further, a sort of unexplained “treatment paradox” has been observed in the current study. The rate of BB and ACE inhibitors use at admission (41% and 91.5%, respectively) declined at discharge (23% and 83%, respectively) among women. To a less extent a similar trend has also been observed among men. Therefore, the impact of this treatment paradox needs further evaluation.

In our study, women presented with AMI are not only having a downward trend in the use of fibrinolytic therapy but also have declined interventional management. This low rate of cardiac catheterization and PCI in women is consistent with several reports from different ethnicities [3], [7], [37], [43]. The rate of diagnostic angiography and angioplasty was greater in men (p = 0.001), whereas the rate of stent insertion was comparable in both sexes (P = 0.90) in the present study. However, the overall rate of invasive procedures was very low which is consistent with previous reports from our region [44].

Several investigators reported controversial findings for the hospital outcome in women with unstable ACS after invasive reperfusion therapy [45]–[48]. According to ACC/AHA guidelines in 2007, women with higher risk of unstable ACS appeared to benefit from early intervention. Whereas, no direct benefit from invasive strategy for low risk features (i.e., without elevated markers or ST depression) was reported [30]–[31]. A recent study advocates an early invasive strategy in women on the same principles as in men [16].

Under treatment of women presenting with ACS is likely multifactorial [33]. This could be in part explained by the delay in symptom recognition and initial assessment, lack of participation in clinical trials and fear of complications.

***In summary***, the present analysis is the first report from a small developing country in the Middle East that describes the epidemiology and trends of ACS over a 20- year period. Our study confirms that women presenting with ACS are characterized with worse clinical profiles, undertreatment and higher mortality rate regardless of age in comparison to men. The novel finding in the current study is the significant improvement in the mortality among females despite the substantial increase in the number of females admitted with ACS over the last 2 decades. It is useful to highlight the different pathophysiology of ACS in females based on their age. Further, the study addresses the relationship of adherence to the use of evidence based therapy and the improvement of outcome in women presenting with ACS.

### Limitation of the Study

Our study is constrained by the limitations inherent to its observational design. Inaccuracies in the diagnosis and coding of ACS in routine data are well recognized, so we have to rely on the accuracy of such data. Temporal changes in referral and coding practices, diagnostic accuracy, and awareness of ACS as a diagnostic entity may have influenced our findings. Presence of missing data or measurement errors and possible confounding by variables not controlled for also adds to our limitations. Furthermore, our study did not address the angiographic data and long-term outcomes. There have been some changes in treatment options since the data were recorded. The broader applicability of our findings to other countries or regions is questionable. A recent report from Qatar showed that the unique social, cultural, religious, environmental, and economic factors are influencing the healthy lifestyle of the Qatari women [49].

### Conclusions

Gender discrepancy exists in many aspects of ACS patients and the mortality rate was higher in women of all age groups, which may be due to underestimation of patient risk. However, a significant improvement in the outcome of ACS patients, particularly women has been observed over the study period. Greater awareness of the treatment risk paradox may help to eliminate the gender gap between current guidelines and management practices and may potentially help to improve short-term outcome. Although, substantial improvement in the hospital outcome has been noticed, guidelines adherence and improvement in the hospital care have not been optimized yet.
